# Two new non-spiny *Solanum* (Solanaceae) from the Gran Chaco Americano and a key for the herbaceous glandular-pubescent solanums from the region

**DOI:** 10.3897/phytokeys.74.10159

**Published:** 2016-11-08

**Authors:** Tiina Särkinen, Sandra Knapp

**Affiliations:** 1 Royal Botanic Garden Edinburgh, 20A Inverleith Row, EH3 5LR Edinburgh, United Kingdom; 2 Department of Life Sciences, Natural History Museum, Cromwell Rd, SW7 5BD London, United Kingdom

**Keywords:** Bolivia, chaco woodlands, endemism, Morelloid clade, South America, Solanaceae, *Solanum* section *Solanum*

## Abstract

The Gran Chaco Americano is a major savanna woodland system in South America that harbours great plant and animal diversity. Two new herbaceous species of the Morelloid clade of *Solanum* (largely corresponding to the traditional Solanum section Solanum) are described here from the Bolivian Chaco. Both species are morphologically similar to a group of related species with glandular pubescence and enlarged, foliaceous calyces that includes *Solanum atriplicifolium* Gillies ex Nees, *Solanum nitidibaccatum* Bitter, *Solanum physalifolium* Rusby, *Solanum sarrachoides* Sendtn. and *Solanum tweedianum* Hook. *Solanum woodii* Särkinen & S.Knapp, **sp. nov.** is unusual in the Morelloid clade in having tapering anthers on short filaments, and is superficially similar to the unrelated *Solanum anomalostemon* S.Knapp & M.Nee from dry forests in Peru. *Solanum michaelis* Särkinen & S.Knapp, **sp. nov.** is distinct in its enlarged calyx with a short tube and long lobes that apparently reflex at fruit maturity. Both new taxa are illustrated, their conservation status assessed, and their distributions mapped. We also provide a key to the glandular-pubescent herbaceous *Solanum* species of the Chaco vegetation to aid in identification of these taxa.

## Abstract (es)

El Gran Chaco americano es un ecosistema importante en América del Sur de bosques de sabana que alberga una gran diversidad de plantas y animales. Se describen aquí dos especies nuevas de plantas herbáceas del chaco boliviano pertenecientes al clado Morelloid de *Solanum* (en gran parte correspondiente a la tradicional *Solanum* sección *Solanum*). Ambas especies son morfológicamente similares a un grupo de especies relacionadas, que poseen pubescencia alargada y glandular, además de cálices foliáceos que incluye *Solanum atriplicifolium* Gillies ex Nees, *Solanum nitidibaccatum* Bitter, *Solanum physalifolium* Rusby, *Solanum sarrachoides* Sendtn. y *Solanum tweedianum* Hook. *Solanum woodii* Särkinen & S.Knapp, **sp. nov.** es inusual en el clado Morelloid en poseer anteras cónicas y filamentos cortos, además es superficialmente similar a la no relacionada *Solanum anomalostemon* S.Knapp & M.Nee de los bosques secos de Perú. *Solanum michaelis* Särkinen & S.Knapp, **sp. nov.** es distinta en el cáliz alargado con un tubo corto y lóbulos largos que aparentemente se doblan hacia atrás en la madurez del fruto. Se presentan aquí ilustraciones de las dos nuevas especies, se evalua su estado de conservación, y se proporcionan mapas de su distribución. También se proporciona una clave para las especies herbáceas de *Solanum* del Chaco con pubescencia glandular para contribuir a la identificación de estas especies.

## Introduction

*Solanum* L. is one of the most species-rich vascular plant genera in South America (Jørgensen et al. 2011), where many new species continue to be described (e.g., Anderson et al. 2006; Stern 2014; Stern and Bohs 2010; Knapp 2010a,b; Farruggia and Bohs 2010; Tepe and Bohs 2009; Tepe et al. 2012; Särkinen et al. 2013a, 2015a, 2015b). Within South America, the tropical Andes represent one of the main centres of species diversity for *Solanum* for both spiny (Leptostemonum clade, see Stern et al. 2011) and non-spiny clades (Weese and Bohs 2007; Särkinen et al. 2013b). Other centres of diversity include dry regions such as the Atacama desert (Regmandra clade, Bennett 2008), and seasonally dry tropical forests including the Gran Chaco Americano (Cyphomandropsis clade, Bohs 2001; Dulcamaroid clade, Knapp 2013; Geminata clade, Knapp 2002, 2008; section Gonatotrichum, Stern et al. 2013; section Erythrotrichum, Agra 2008).

The Gran Chaco Americano is the most extensive dry forest complex in the Americas and the second largest forested lowland area in South America after the Amazon (Galera and Ramella 1997, Olson et al. 2000). The ecoregion covers 1,100,000 km^2^ in eastern Bolivia (19% of the total area of Gran Chaco) and northern Argentina (46%), all of western Paraguay (34%), and a small portion of Brazil (1%) (Galera and Ramella 1997; Olson et al. 2000). Two sub-regions can be recognized: a) the Dry Chaco where the dominant vegetative structure is xerophytic deciduous forest with multiple layers including a canopy, sub-canopy, shrub and herbaceous layer; and b) the Humid Chaco composed of seasonally flooded plains covered by wetlands and palm tree savannas. Some authors distinguish seasonally dry tropical forests from Chaco vegetation per se (Pennington et al. 2006), but here we are using a more inclusive categorization. The Chaco represents one of the last great undisturbed areas of habitat in South America outside Amazonia, but recent rates of habitat conversion are alarmingly high (Huang et al. 2009; Hoyos et al. 2013; Yanosky 2013). The region is poorly explored, but rich in diversity that has been shown to be in rapid decline (Periago et al. 2014). Plant collections from the Chaco region are poorly represented relative to other habitats in the relevant countries; for example, of the 17,961 Paraguayan plant specimens held in the collections of the Natural History Museum
(BM), only 0.6% are from the Chaco ecoregion. New collections made from the region are of interest not only for documenting the diversity of this under-collected and highly threatened area (see Galera and Ramella 1997, http://www.worldwildlife.org/ecoregions/nt0210), but also because novelties are likely to be found.

The Morelloid clade is a group of ca. 75 species most of which are endemic to the tropical Andes (Bohs 2005; Särkinen et al. 2015c). The clade includes five major groups traditionally recognised at the sectional level (sections *Solanum*, *Campanulisolanum* Bitter, *Parasolanum* A.Child pro parte, *Chamasarachidium* Bitter, and *Episarcophyllum* Bitter), which are in the process of re-circumscription based on molecular results (Särkinen et al. 2015c). Section *Solanum* is the largest of these with ca. 52 species and ca. 580 published names and is the only group to occur outside of the Americas. Section *Solanum* is distinguished by its herbaceous or sub-shrubby habit, inflorescences usually positioned along the internodes, small flowers and fruits, and the usual possession of stone cells in the fruits (Bitter 1911), which appear as small, seed-like structures that are usually white and spherical rather than flattened and brown or yellowish brown like the seeds. These stone cells are derived from accretions of sclerenchyma in the mesocarp (Bitter 1911, 1914; Danert 1969). Although some studies have been done to clarify the taxonomy of the Old World and North American species of the Morelloid group (Edmonds 1977, 1978; Schilling 1981), monographic study is needed to aid species identification and to clarify synonymy, especially in Andean South America where most of the species diversity is found (Edmonds 1972; Barboza et al. 2013) and where the Morelloid clade is amongst the most diverse groups of *Solanum*.

Recent taxonomic work focusing on delivering a global monographic treatment of the Morelloid clade has resulted in the description of various new species from the tropical Andes (Särkinen et al. 2013a, 2015a, 2015b). A further two new species are here described based on morphological and preliminary molecular data from Bolivian Chaco woodlands. A total of six species of *Solanum* from the Morelloid clade are now known to occur in the Chaco region, and we provide a key for the identification of similar glandular-pubescent herbaceous non-spiny solanums from the Gran Chaco Americano.

## Materials and methods

Descriptions are based on field work and examination of herbarium specimens from K, LPB, MO, and NY (acronyms follow Index Herbariorum; http://sweetgum.nybg.org/science/ih/). Many more duplicates of the specimens cited collected by M. Nee and J.R.I. Wood are expected to be found in Bolivian (USZ for Nee, BOLV and USZ for Wood) and other herbaria deposited under *Solanum* sp. or *Solanum physalidicalyx* Bitter.

Specimens with coordinates were mapped directly and those lacking coordinates were located using Google Earth and gazetteers. Extent of Occurrence (EOO) and Area of Occupancy (AOO) were calculated using GeoCat (www.geocat.kew.org) with a 2 km cell width for AOO calculation. The preliminary conservation status of each species was assessed using the IUCN (2014) criteria based on the GeoCat analyses (Bachman et al. 2011) combined with field knowledge. All specimens are cited in the text, and full data is provided in the Suppl. material 1 and on Solanaceae Source (www.solanaceaesource.org).

## Taxonomic treatment

### Solanum michaelis

Taxon classificationPlantaeSolanalesSolanaceae

Särkinen & S.Knapp sp. nov.

urn:lsid:ipni.org:names:77158524-1

Fig. 1

#### Diagnosis.

Like *Solanum sarrachoides* Sendtn. and *Solanum physalifolium* Rusby but differing in having larger anthers 2.5–3.2 mm long, and similar to *Solanum tweedianum* Hook. in having long calyx lobes but differing in having a shorter calyx tube in both flower (0.8–1.3 mm) and fruit (2.0–2.5 mm).

#### Type.

**Bolivia. Tarija**: Prov. Gran Chaco, 44.5 km (by rd) W from upper bridge over Rio Pilcomayo and 17.7 NE of Palos Blancos, on rd from Villa Montes to Palos Blancos, 21°27'S, 63°40'45"W, 815 m, 21 Mar 2007 (fl,fr), *M. Nee & R. Flores S. 54821* (holotype: LPB; isotypes: BM [BM001211859], MO [sheet number 6073914, barcode MO-2113149], NY [NY00853628], UT [UT-126715]; [records indicate that duplicates were sent to CAS, CORD, G, MEXU, NSW, SI, USZ, US, UT, WIS]).

#### Description.

Decumbent to erect subwoody herb to 1 m tall, spreading to up to 2 m in diameter. Stems 3–4 mm in diameter at base, spreading or erect, terete, straw coloured, glabrescent; new growth densely glandular-papillate and pubescent with a mixture of patent, simple, uniseriate eglandular and glandular trichomes, the trichomes of several lengths, 1-celled to 17-celled, 0.2–2 mm long, translucent, if glandular then with a terminal gland (this often breaking off). Sympodial units difoliate, not geminate. Leaves simple, (2.4–)4.0–7.6 cm long, (1.4–)2.3–3.0(–4.0) cm wide, ovate; adaxial surface moderately pubescent with both eglandular and glandular hairs along lamina and veins; abaxial surface more densely pubescent along veins; major veins 3–5 pairs; base truncate to rounded; margins entire to shallowly and unevenly lobed (mostly near the base); apex acute; petiole (0.7–)1.5–2.0 cm long, pubescent with spreading eglandular and glandular hairs like those on the stem. Inflorescences 2.5–3.5 cm long, lateral, internodal to leaf-opposed, simple, racemose, with (6–)7–10(–12) flowers, pubescent with both eglandular and glandular trichomes like those on stem; peduncle 1.4–3.3 cm long; pedicels spaced 0–1 mm apart, 6–10 mm long, ca. 0.2 mm in diameter at base and apex, straight and spreading at anthesis, articulated at the base. Buds ellipsoid, white or purple-tinged, densely pubescent with spreading, multicellular hairs (see under calyx), the corolla not strongly exerted from the calyx, exceeding the calyx lobes by less than ½ of their lenghts before anthesis. Flowers 5-merous, all perfect. Calyx tube 0.8–1.3 mm long, the lobes 1.4–3.7 mm long, 0.6–1.0 mm wide, triangular with long-acuminate apices, densely pubescent with both eglandular and glandular trichomes, the eglandular trichomes 1.5–3.5 mm long. Corolla 0.7–1.3 cm in diameter, white with a green-black basal central star, stellate, lobed 1/2 way to the base, the lobes 2.5–3.2 mm long, 1.5–2.5 mm wide, reflexed at anthesis, later spreading, sparsely pubescent abaxially with multicellular simple spreading eglandular uniseriate trichomes to 0.5 mm long, densely papillate on the tips and margins. Stamens equal; filament tube 0.1–0.25 mm long; free portion of the filaments 0.2–0.3 mm long, adaxially pubescent with tangled eglandular simple uniseriate trichomes; anthers 2.5–3.2 mm long, 0.9–1.1 mm wide, ellipsoid, yellow, poricidal at the tips, the pores lengthening to slits with age. Ovary subglobose, glabrous; style 4–5 mm long, exerted 1.5–2.0 mm beyond the anther cone, densely pubescent with 4-celled simple uniseriate trichomes in the basal ½ or 3/5 where included in the anther cone; stigma capitate, the surface minutely papillate. Fruit a subglobose berry, slightly flattened, 5–12 mm in diameter, green and mottled with white vein-like reticulations (black when ripe fide *Fuentes & Navarro 2607*), the surface of the pericarp shiny; fruiting pedicels 1.6–2.0 mm long, ca. 0.5 mm in diameter at the base, ca. 1.0 mm in diameter at the apex, spaced 1–2 mm apart, strongly recurving, dropping off with the fruit leaving raised pedicels scars to 0.1 mm high; fruiting calyx tube 2.0–2.5 mm long, the lobes 5–8 mm long and 3.0–3.5 mm wide, spreading to reflexed. Seeds 15–25 per berry, 1.7–2.0 mm long, 1.1–1.5 mm wide, tear-drop shaped, pale brown, the surface minutely pitted, the hilum positioned subapically, the testal cells pentagonal in outline. Stone cells absent.

**Figure 1.**
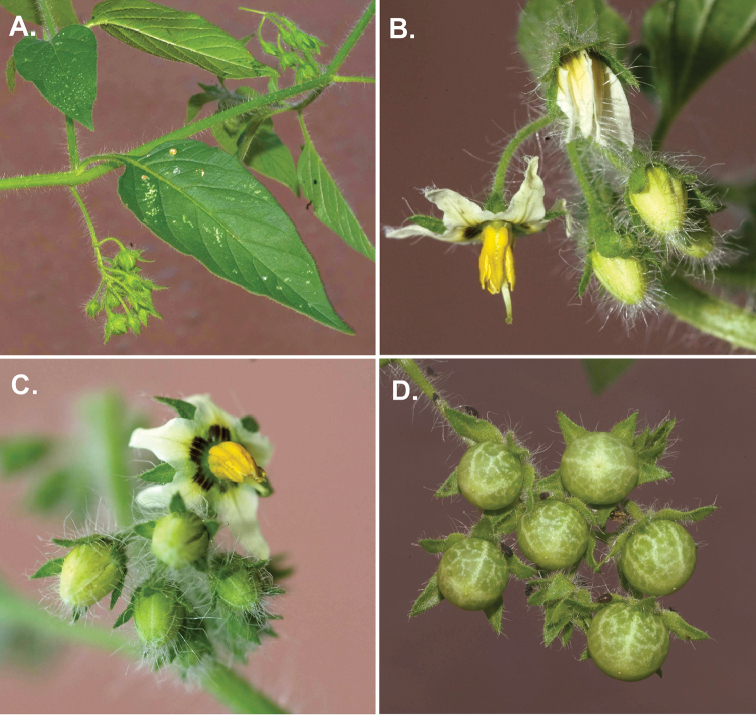
*Solanum michaelis*. **A** Fruiting stem **B** Inflorescence with details of indumentum of simple, multi-cellular eglandular and glandular trichomes along the stem, calyx and corolla **C** Flower at full anthesis with buds **D** Maturing fruit (**A–D** *Nee & Flores 54821*: photos by Michael Nee).

#### Distribution

**Figure 2.**
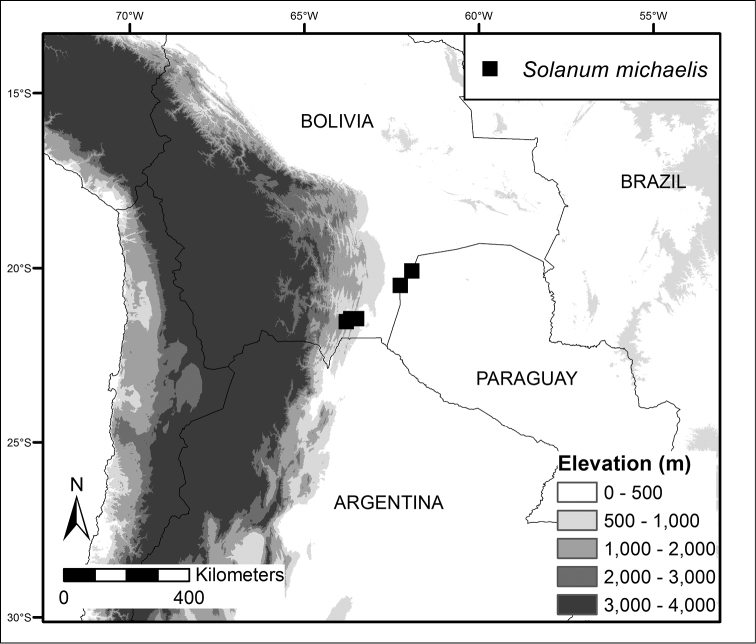
Distribution map of *Solanum michaelis*.

(Figure 2). Endemic to Bolivia in the Departments of Tarija and Santa Cruz; although the species is to be expected in adjacent Paraguay. *Solanum michaelis* grows in dry Chaco vegetation and in lower inter-Andean valleys, along slopes in sandy soils in mostly unshaded dry creek beds on bare soil, often in areas that have been burned, or in more humid Chaco vegetation at the edge of “palmares” (stands of *Copernicia alba* Morong) between 300–900 m elevation.

#### Ecology.

Flowering in March and between June and September, fruiting from June to September probably toward the end of the rainy season (Jan-Apr) and then sporadically with occasional rains during the dry season.

#### Etymology.

The species epithet honours Dr Michael Nee, whose collections from Bolivia have provided the much needed material to complete descriptions of many recently published new species within *Solanum*, including the two described here. His collections and taxonomic work over the past 50 years have contributed to the understanding of morphological diversity of *Solanum*. His taxonomic work in the genus has been fundamental in resolving and typifying the 6,967 published names of *Solanum*.

#### Conservation status.

The preliminary IUCN (IUCN 2014) threat status of *Solanum michaelis* is Endangered (EN) based on the small known extent of the species occurrence (EOO=2,716 km^2^) as well as the extremely small observed area of occupancy (AOO=20 km^2^). Although collection densities in the tropical Andes remain low, the very narrow distribution of the new species suggests conservation concern, because *Solanum michaelis* is likely to be highly vulnerable to grazing pressure and changes in rainfall patterns due to its ephemeral ecology. The Chaco woodlands in Bolivia and Paraguay are highly threatened by land use change due to agricultural expansion and logging (Huang et al. 2009). Two populations of *Solanum michaelis* are known to occur within the protected area network in Bolivia, one in the Parque Nacional de Gran Chaco Kaa-lya along the border with Paraguay, and another in the Parque Nacional de Serranía del Aguaragüe.

#### Additional specimens examined.

**Bolivia. Santa Cruz**: **Prov. Cordillera**, Parque Nacional Kaa-Iyá del Gran Chaco, hito 27 de noviembre, 20°05'16"S, 61°55'19"W, 320 m, 17 Jul 1998 (fl,fr), *A. Fuentes 2607* (NY); alrededor del pueblo de la Brecha, 22 May 1999 (fl,fr), *R. Chávez de Michel 2677* (LPB, NY); 4 km de Puerto Guaraní, al N frontera Paraguaya, 20°30'S, 62°15'W, 400 m, 19 Jun 1992 (fl,fr), *B. Mostacedo 380* (MO). **Tarija**: **Prov. Gran Chaco**, 10 km S de Palmar Grande, camino Yacuiba-Villa Montes, 10 Sep 1977 (fl,fr), *A. Krapovickas 31088* (MO); 2 km N de Palmar Grande, 38 km S de Villa Montes, 21°27'S, 63°30'W, 400 m, 10 Sep 1977 (fl), *A. Krapovickas 31137* (K, MO); 0.5 km E of Chuvere, 21°32'15"S, 63°48'10"W, 870 m, 23 Mar 2007 (fl,fr), *M. Nee 54876* (MO, NY).

#### Discussion.

*Solanum michaelis* differs from the co-occurring and morphologically similar *Solanum sarrachoides* and the higher elevation yungas species *Solanum physalifolium* in having larger anthers (2.5–3.2 mm long), while both *Solanum sarrachoides* and *Solanum physalifolium* have anthers < 2.2 mm long. *Solanum physalifolium* has similar shiny green-mottled berries, but occurs at higher elevations (1,400–2,900 m) in yungas or wet forest vegetation and has broadly ovate calyx lobes that partially enclose the fruit at maturity. *Solanum tweedianum* has similar sized anthers but a longer calyx tube (ca. 1.5–2.0 mm in flower and to ca. 5 mm or more in fruit) which fully encloses the berry both during development and at fruit maturity (Barboza et al. 2013). *Solanum michaelis* has similarly long calyx lobes but a shorter calyx tube in both flower (0.8–1.3 mm) and fruit (2.0–2.5 mm) that does not enclose the fruit and appears to sometimes have reflexed calyx lobes at fruit maturity (e.g., *Fuentes & Navarro 2607*).

### Solanum woodii

Taxon classificationPlantaeSolanalesSolanaceae

Särkinen & S.Knapp sp. nov.

urn:lsid:ipni.org:names:77158525-1

Fig. 3

#### Diagnosis.

Similar to *Solanum tweedianum* Hook., but differing in having shorter calyx lobes in flower (1.2–2.1 mm) and fruit (2.0–3.5 mm) and broadly ovoid anthers, and to *Solanum physalifolium* Rusby but differing in having long-triangular calyx lobes, shorter filaments 0.1–0.4 mm long, and broadly ovoid anthers.

#### Type.

**Bolivia. Santa Cruz**: Prov. Valle Grande, pasando el puente Santa Rosa, a 78 km desde Serrano hacia Valle Grande, 18°42.483'S, 64°17.585'W, 1169 m, 4 Apr 2003 (fl,fr), *J.R.I. Wood 19616* (holotype: LPB).

#### Description.

Decumbent, slender annual (fide labels) herb to 30–40 cm. Stems 1.0–5.0 mm in diameter, terete, much branching, pale yellow or greenish beige, glabrescent; new growth densely pubescent with spreading translucent 5–8-celled simple uniseriate glandular trichomes c. 0.5 mm long, some to 1 mm. Sympodial units difoliate, not geminate. Leaves simple, (2.3–)4.5–8.0 cm long, (1.5–)2.2–4.3 cm wide, elliptic to ovate, thin-membranous; adaxial surface moderately pubescent with spreading hairs as on stem evenly spaced along lamina and veins; abaxial surface more densely pubescent along veins; major veins 5–7 pairs; base attenuate to decurrent; margins entire to shallowly and unevenly toothed, the lobes narrow; apex acute; petiole 0.8–4.5 cm long, sparsely pubescent with simple 5–8-celled uniseriate trichomes like those of the stems. Inflorescences 1.5–3.0 cm long, simple, opposite the leaves, with (2–)3–7 flowers, sparsely pubescent with simple 5–8-celled uniseriate trichomes like those of the stems; peduncle 0.9–1.8 cm long, ca. 0.3 mm in diameter at the apex and ca. 0.5 mm in diameter at the base; pedicels spaced 0–1 mm apart, 0.7–1.1 cm long, ca. 0.2 mm in diameter at the base and ca. 0.3 mm in diameter at the apex, straight and spreading at anthesis, articulated at the base. Buds ovoid, white, the corolla strongly exerted from the calyx before anthesis, exceeding the lobes by up to two times their length. Flowers 5-merous, all perfect. Calyx tube 0.6–0.7 mm long, the lobes 1.2–2.1 mm long, 0.8–1.0 mm wide, ovate to elliptic in outline with acute apices, somewhat spreading at anthesis, sparsely pubescent with simple 5–8-celled uniseriate glandular trichomes like those of the stems. Corolla 1.0–1.5 cm in diameter, white with a greenish-purple central star at the base, stellate, lobed to the middle, the lobes 4.0–6.0 mm long, 2.0–3.0 mm wide, reflexed at anthesis, sparsely pubescent abaxially with very short 1–2-celled simple uniseriate eglandular trichomes. Stamens equal; filament tube ca. 0.5 mm long; free portion of the filaments 0.1–0.4 mm long, adaxially pubescent with 4–7-celled uniseriate eglandular trichomes; anthers (2.5–)3.0–3.8 mm long, 1.2–1.4 mm wide at base, ca. 0.5 mm at tip, tapering and narrowly triangular to triangular in outline, yellow, poricidal at the tips, the pores lengthening to slits with age. Ovary globose, glabrous; style 4.5–5.0 mm long, exerted 1.5–2.0 mm beyond the anther cone, curved at the very tip, densely pubescent with 2–3-celled simple uniseriate trichomes in the basal 1/3 where included in the anther cone; stigma minutely capitate, the surface papillate. Fruit a globose berry, 5–9 mm in diameter, green (immature), the pericarp thick and shiny; fruiting pedicels 0.7–1.0 cm long, ca. 0.5 mm in diameter at the base, ca. 0.6 mm in diameter at the apex, spaced 0–1 mm apart, spreading to recurved; fruiting calyx tube ca. 1 mm long, the lobes 2.0–3.5 mm long, spreading to reflexed. Seeds 15–30 per berry, 1.6–2 mm long, 1–1.5 mm wide, flattened, teardrop-shaped with a subapical hilum, yellow, the surface minutely pitted, the testal cells pentagonal in outline with the lateral cell walls elongate and the seeds from mature fruits appearing hairy. Stone cells absent.

**Figure 3.**
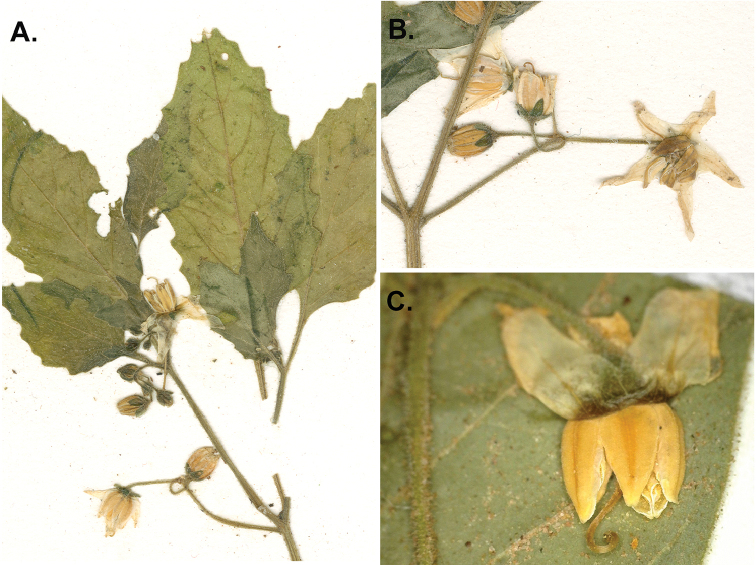
*Solanum woodii*. **A** Flowering stem **B** Inflorescence with details of buds, calyx and corolla **C** Flower at full anthesis (**A–B** *Wood 21787*; **C** *Nee et al. 51967*; photos by Gwen Davis).

#### Distribution

**Figure 4.**
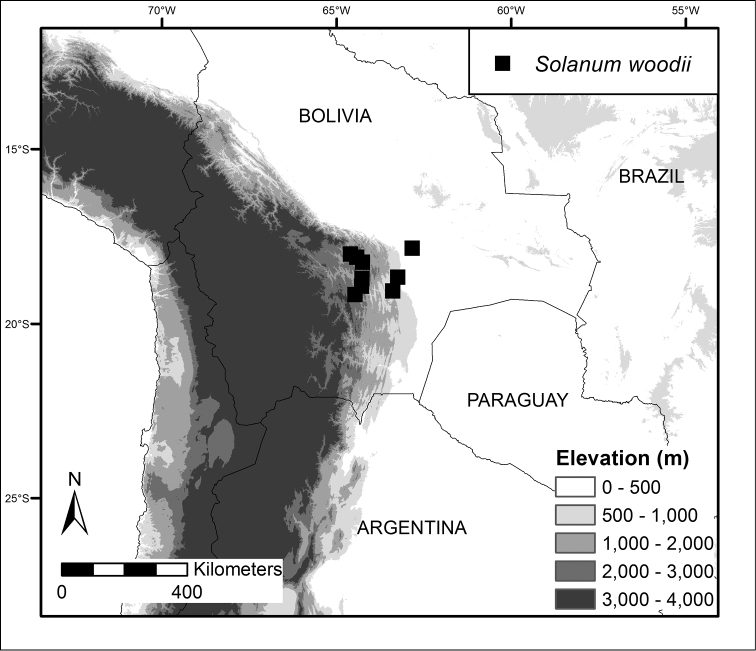
Distribution map of *Solanum woodii*.

(Figure 4). Endemic to Bolivia in the Departments of Chuquisaca and Santa Cruz, growing in Chaco and Chaco forests of inter-Andean valleys, in dry Chaco woodlands on sandy and clay soils near water sources, rivers and in moist depressions in partial or full shade; between 300–1,800 m elevation.

#### Ecology.

Flowering and fruiting during the wet season from January–April.

#### Etymology.

The species epithet honours John R.I. Wood who has collected extensively in central and eastern Bolivia and mentored numerous young Bolivian botanists. Material collected by John throughout his career has been the basis for the description of many new species, and here we add yet another to that long list.

#### Conservation status.

The preliminary IUCN (IUCN 2014) threat status of *Solanum woodii* is Vulnerable (VU, B1) based on the small extent of occurrence (EOO=19,656 km^2^). The area of occupancy is even smaller (AOO=36 km^2^) and would merit status as endangered (EN), but knowing that collection densities in this part of south-central Bolivia remain low and that the collections are mainly along the sparse road network, we prefer basing our assessment on the extent rather than area of occurrence. No occurrences are known within protected areas in Bolivia thus far.

#### Additional specimens examined.

**Bolivia**. **Chuquisaca**: **Prov. Belisario Boeto**, bajando de Nuevo Mundo hacia Santa Rosa, en dirección al Río Grande, 18°55'37"S, 64°17'07"W, 1,350 m, 2 Mar 2006 (fl,fr), *J.R.I. Wood 22341* (K). **Santa Cruz**: **Prov. Andrés Ibáñez**, 5–8 km E-SE of Comunidad Don Lorenzo, nr Quebrada Caracoré, on rd to Estancia Caracoré, La Bola area, 17°50'S, 62°50'W, 310 m, 4 Jan 1996 (fl,fr), *M. Nee 46692* (MO, NY). **Prov. Caballero**, El Canal, a 7 km N de Saipina, 18°00'20"S, 64°36'15"W, 1,500 m, 31 Mar 1994 (fl,fr), *J. Balcázar 368* (MO); cerca de Pulquina hacia el Río Mizque, ca. 2 km antes de llegar al primer cruce de río y ca. 1 km de la comunidad Witron, 18°05'57"S, 64°25'52"W, 1,562 m, 20 Feb 2007 (fl,fr), *J.R.I. Wood 22840* (K). **Prov. Cordillera**, along gravel rd eastward, just E of new highway from Santa Cruz to Abapó, the turnoff 3 km N of bridge over Río Seco, 18°63'33"S, 63°23'33"W, 510 m, 19 Apr 1998 (fl), *M. Nee 49056* (MO); floodplain and adjacent upland along the Río Seco, 1.5 km NW of highway at the town of Río Seco along rd to La Florida, 18°40'S, 63°15'W, 525 m, 24 Mar 2002 (fl), *M. Nee 51967* (MO). **Prov. Vallegrande**, steep slopes of valley of the Río Grande, on rd from Pucará to the new bridge over the Río Grande, 10 km (by winding road) NW of Puente Santa Rosa, 18°70'00"S, 64°28'33"W, 1,500 m, 29 Jan 1994 (fl), *M. Nee 44742* (MO); bajando de Pucará hacia Santa Rosa del Río Grande, 18°14'43"S, 64°16'00"W, 1,787 m, 6 Mar 2005 (fl,fr), *J.R.I. Wood 21787* (K); La Higuera, bajando de Pucará al puente de Santa Rosa, 18°42'06"S, 64°16'23"W, 1,580 m, 18 Feb 2007 (fl,fr), *J.R.I. Wood 22790* (K).

#### Discussion.

*Solanum woodii* is unusual in having tapering, somewhat cone-shaped anthers with a beak-like tip (see Fig. 1C); this character, however, can be difficult to see in older flower with dehisced anthers. Amongst other glandular-viscid herbaceous solanums it could be confused with *Solanum tweedianum* and *Solanum physalifolium*. *Solanum woodii* is sympatric with *Solanum tweedianum* but the latter species has longer calyx lobes in flower (3.5–5(–7) mm) and fruit (>5 mm), and slightly larger ellipsoid anthers (3.0–)4.0–4.5 mm long that are rectangular in outline (equally wide along their entire length) rather than broadest at the base; the calyx of *Solanum tweedianum* is accrescent and completely covers the berry at maturity, while that of *Solanum woodii* is spreading and does not become accrescent.

The unusual anther shape in *Solanum woodii* resembles that of the enigmatic *Solanum anomalostemon* S.Knapp & M.Nee described from the dry inter-Andean valley of the Rio Apurimac in southern Peru (Knapp and Nee 2009). *Solanum anomalostemon* is morphologically unique within *Solanum* in having cordate anthers, and was thought to belong to the Morelloid clade (Knapp and Nee 2009). Recent molecular phylogenetic evidence, however, showed it belongs to the Mapiriense clade (Särkinen et al. 2015c), along with a small group of species that have similar tapering anthers (see Bohs 2005). Despite the similarity in anther shape, preliminary molecular data suggest *Solanum woodii* is a member of the Morelloid clade rather than closely related to *Solanum anomalostemon* and other members of the Mapiriense clade (T. Sarkinen, unpubl. data).

### Key to glandular-pubescent herbaceous solanums in Chaco vegetation

**Table:** 

1	Anthers 0.8–2.1 mm long	**2**
–	Anthers 2.5–5.0 mm long	**3**
2	Calyx lobes completely enclosing the corolla in bud; inflorescences with flowers clustered near the tips; leaf base truncate	***Solanum sarrachoides***
–	Calyx lobes not completely enclosing the corolla in bud; inflorescences with flowers spaced 1–3 mm apart along the rachis; leaf base rounded to cuneate	***Solanum nitidibaccatum***
3	Anthers 2.5–3.2(-3.8) mm long; calyx (tube and/or lobes) covering 0–20% of the berry in fully mature fruits	**4**
–	Anthers (3.8-)4.0–5.0 mm long; calyx (tube and/or lobes) covering at least 50% of the berry in fully mature fruits	**5**
4	Calyx with spreading trichomes 1.5–3.5 mm long; anthers ellipsoid, rectangular in outline; fruiting pedicels spaced (0-)1–2 mm apart	***Solanum michaelis***
–	Calyx with spreading trichomes 0.5–1.0 mm long; anthers conical, triangular in outline; fruiting pedicels spaced 0–1 mm apart	***Solanum woodii***
5	Calyx lobes 2.5–3.0 mm long in flower; fruiting calyx not markedly enlarged and inflated, the calyx tube slightly growing but neither tube nor lobes accrescent; fruit often slightly visible or calyx tube reaching just beyond the top of the berry	***Solanum atriplicifolium***
–	Calyx lobes 3.5–5(-7) mm long in flower; fruiting calyx markedly enlarged and inflated, both calyx tube and lobes accrescent; fruit fully covered by calyx tube	***Solanum tweedianum***

## Supplementary Material

XML Treatment for Solanum michaelis

XML Treatment for Solanum woodii

## Supplementary materials

Supplementary material 1 Occurrence records Data type: Excel csv file Explanation note: Occurrence records of the two new *Solanum* species. This dataset is made available under the Open Database License (http://opendatacommons.org/licenses/odbl/1.0/). The Open Database License (ODbL) is a license agreement intended to allow users to freely share, modify, and use this Dataset while maintaining this same freedom for others, provided that the original source and author(s) are credited.Tiina Särkinen, Sandra Knapp

## References

[B1] Agra M (2008) Four new species of Solanum section Erythrotrichum (Solanaceae) from Brazil and Peru, and a key to the species of the section. Systematic Botany 33: 556–565. doi: 10.1600/036364408785679897

[B2] Anderson GJ Prohens J Nuez F Martine C (2006) *Solanum perlongistylum* and *S. catilliflorum*, new endemic Peruvian species of *Solanum*, section Basarthrum, are close relatives of the domesticated pepino, *S. muricatum*. Novon 16(2): 161–167. doi: 10.3417/1055-3177(2006)16[161:SPASCN]2.0.CO;2

[B3] Bachman S Moat J Hill A de la Torre J Scott B (2011) Supporting Red List threat assessments with GeoCAT: geospatial conservation assessment tool. ZooKeys 150: 117–126. doi: 10.3897/zookeys.150.2109 10.3897/zookeys.150.2109 PMC3234434 22207809

[B4] Barboza GE Knapp S Särkinen T (2013) *Solanum* Grupo VII. Moreloide. In: Barboza GE (Ed.) Flora Argentina: Flora Vascular de la Republica Argentina, Dicotyledoneae, Solanaceae Vol. 13. Instituto de Botanica Darwinion, San Isidro, Argentina, 231–264.

[B5] Bennett JR (2008) Revision of Solanum section Regmandra (Solanaceae. Edinburgh Journal of Botany 65: 69–112. doi: 10.1017/S0960428608004903

[B6] Bitter G (1911) Steinzellkonkretionen im Fruchtfleisch beerentragender Solanaceen und deren systematische Bedeutung. Botanische Jahrbücher für Systematik, Pflanzengeschichte und Pflanzengeographie 45: 483–507.

[B7] Bitter G (1914) Weitere Untersuchungen über das Vorkommen von Steinzellkonkretionen in Fruchtfleisch beerentragender Solanaceen. Abhandlungen Naturwissenschaften Vereine Bremen 23: 114–163.

[B8] Bohs L (2001) Revision of Solanum section Cyphomandropsis (Solanaceae). Systematic Botany Monographs 61: 1–85. doi: 10.2307/25027891

[B9] Bohs L (2005) Major clades in *Solanum* based on *ndhF* sequence data. In: Keating R Hollowell VC Croat TB (Eds) A festschrift for William G. D’Arcy – The legacy of a taxonomist. Missouri Botanical Garden Press, St. Louis, 27–49.

[B10] Danert S (1969) Über die Entwicklung der Steinzellkonkretionen in der Gattung *Solanum*. Die Kulturplanze 17: 299–311. doi: 10.1007/BF02097952

[B11] Edmonds JM (1972) A synopsis of the taxonomy of Solanum sect. Solanum (Maurella) in South America. Kew Bulletin 27: 95–114. doi: 10.2307/4117874

[B12] Edmonds JM (1977) Taxonomic studies on Solanum section Solanum (Maurella). Botanical Journal of the Linnean Society 75: 141–178. doi: 10.1111/j.1095-8339.1977.tb01482.x

[B13] Edmonds JM (1978) Numerical taxonomic studies on Solanum L. section Solanum (Maurella). Botanical Journal of the Linnean Society 76: 27–51. doi: 10.1111/j.1095-8339.1978.tb01497.x

[B14] Farruggia F Bohs L (2010) Two new South American species of Solanum section Crinitum (Solanaceae). PhytoKeys 1: 67–77. doi: 10.3897/phytokeys.1.661 10.3897/phytokeys.1.661 PMC3174426 22171169

[B15] Galera FM Ramella L (1997) CPD Site SA22: Gran Chaco. In: Davis SD Heywood VH Herrera-MacBryde O Villa-Lobos J Hamilton AC (Eds) Centres of Plant Diversity: a guide and strategy for their conservation. Volume 3: The Americas. World Wide Fund for Nature (WWF and IUCN (World Conservation Union), IUCN Publications Unit, Cambridge, 411–415.

[B16] Hoyos IE Cingolani AM Zak MR Vaieretti MV Goria DE Cabido MR (2013) Deforestation and precipitation patterns in the arid Chaco forests of central Argentina. Applied Vegetation Science 16: 260–271. doi: 10.1111/j.1654-109X.2012.01218.x

[B17] Huang C Kim S Song K Townshend JRG Davis P Altstatt A Rodas O Yanosky A Clay R Tucker CJ Musinsky J (2009) Assessment of Paraguay'S, forest cover change using Landsat observations. Global and Planetary Change 67: 1–12. doi: 10.1016/j.gloplacha.2008.12.009

[B18] IUCN (2014) Guidelines for using the IUCN Red List Categories and Criteria. Version 11. Prepared by the Standards and Petitions Subcommittee http://www.iucnredlist.org/documents/RedListGuidelines.pdf [12Dec2014]

[B19] Jørgensen PM Ulloa Ulloa C León B León-Yánez S Beck SG Nee M Zarucchi JL Celis M Bernal R Gradstein R (2011) Regional patterns of vascular plant diversity and endemism. In: Herzog SK Martínez R Jørgensen PM Tiessen H (Eds) Climate Change and Biodiversity in the Tropical Andes. Inter-American Institute for Global Change Research (IAI) and Scientific Committee on Problems of the Environment (SCOPE), 192–203.

[B20] Knapp S (2002) Solanum section Geminata (G. Don) Walpers (Solanaceae). Flora Neotropica 84: 1–405.

[B21] Knapp S (2008) A revision of the *Solanum havanense* species group (section Geminata (G. Don) Walp. pro parte) and new taxonomic additions to the Geminata clade (*Solanum*: Solanaceae). Annals of the Missouri Botanical Garden 95: 405–458. doi: 10.3417/2006159

[B22] Knapp S (2010a) Four new vining species of *Solanum* (Dulcamaroid Clade) from montane habitats in tropical America. PLoS ONE 5(5): e10502. doi: 10.1371/journal.pone.0010502 20463921 10.1371/journal.pone.0010502 PMC2864764

[B23] Knapp S (2010b) New species of *Solanum* (Solanaceae) from Peru and Ecuador. PhytoKeys 1: 33–51. doi: 10.3897/phytokeys.1.659 10.3897/phytokeys.1.659 PMC3174425 22171167

[B24] Knapp S (2013) A revision of the Dulcamaroid Clade of *Solanum* L. (Solanaceae). PhytoKeys 22: 1–432. doi: 10.3897/phytokeys.22.4041 10.3897/phytokeys.22.4041 PMC3689140 23794937

[B25] Knapp S Nee M (2009) *Solanum anomalostemon* (Solanaceae), an endangered new species from southern Peru with unusual anther morphology. Novon 19: 178–181. doi: 10.3417/2007108

[B26] Olson D Dinerstein E Hedao P Walters S Allnutt T Loucks C Kura Y Kassem K Webster A Bookbinder M (2000) Terrestrial Ecoregions of the Neotropical Realm (map). Conservation Science Program, WWF-US, Washington DC.

[B27] Pennington RT Richardson JE Lavin M (2006) Insights into the historical construction of species-rich biomes from dated plant phylogenies, neutral ecological theory and phylogenetic community structure. New Phytologist 172: 605–616. doi: 10.1111/j.1469-8137.2006.01902.x 17096788 10.1111/j.1469-8137.2006.01902.x

[B28] Periago ME Chillo V Ojeda RA (2014) Loss of mammalian species form the South American Gran Chaco: empty savanna syndrome? Mammal Review 45: 41–53.

[B29] Särkinen TS Gonzáles P Knapp S (2013a) Distribution models and species discovery: the story of a new *Solanum* species from the Peruvian Andes. PhytoKeys 16: 1–20. doi: 10.3897/phytokeys.31.6312 10.3897/phytokeys.31.6312 PMC3881343 24399901

[B30] Särkinen TS Bohs L Olmstead RG Knapp S (2013b) A phylogenetic framework for evolutionary study of the nightshades (Solanaceae): a dated 1000-tip tree. BMC Evolutionary Biology 13: 214. doi: 10.1186/1471-2148-13-214 24283922 10.1186/1471-2148-13-214 PMC3850475

[B31] Särkinen T Gonzáles P Knapp S (2015a) Four new non-spiny *Solanum* (Solanaceae) species from South America. PhytoKeys 44: 39–64. doi: 10.3897/phytokeys.44.8693 10.3897/phytokeys.44.8693 PMC4329390 25698893

[B32] Särkinen T Knapp S Nee M (2015b) Two new non-spiny *Solanum* species from the Bolivian Andes (Morelloid Clade). PhytoKeys 47: 97–109. doi: 10.3897/phytokeys.47.4423 10.3897/phytokeys.47.4423 PMC4389089 25878556

[B33] Särkinen TS Barboza GE Knapp S (2015c) True Black nightshades: Phylogeny and delimitation of the Morelloid clade of *Solanum*. Taxon 64: 945–958. doi: 10.12705/645.5

[B34] Schilling EE (1981) Systematics of Solanum sect. Solanum (Solanaceae) in North America. Systematic Botany 6: 172–185. doi: 10.2307/2418547

[B35] Stern S (2014) A new species of spiny *Solanum* (Solanaceae) from Peru. PhytoKeys 39: 27–34. doi: 10.3897/phytokeys.39.7513 10.3897/phytokeys.39.7513 PMC4152889 25197223

[B36] Stern S Bohs L (2010) Two new species of *Solanum* (Solanaceae) from the Amotape-Huancabamba Zone of southern Ecuador and northern Peru. PhytoKeys 1: 53–65. doi: 10.3897/phytokeys.1.660 10.3897/phytokeys.1.660 PMC3174427 22171168

[B37] Stern S Agra F Bohs L (2011) Molecular delimitation of clades within New World species of the “spiny solanums” (Solanum subg. Leptostemonum). Taxon 60: 1429–1441. 10.1002/tax.605018 PMC7169808 32327851

[B38] Stern S Bohs L Giacomin L Stehmann J Knapp S (2013) A revision of Solanum section Gonatotrichum. Systematic Botany 38: 471–496. doi: 10.1600/036364413X666624

[B39] Tepe EJ Bohs L (2009) Three new species of Solanum section Herpystichum (Solanaceae) from Ecuador. Journal of the Botanical Research Institute of Texas 3: 511–519.

[B40] Tepe EJ Ridley G Bohs L (2012) A new species of *Solanum* named for Jeanne Baret, an overlooked contributor to the history of Botany. PhytoKeys 8: 37–47. doi: 10.3897/phytokeys.8.2101 10.3897/phytokeys.8.2101 PMC3254248 22287929

[B41] Weese TL Bohs L (2007) A three-gene phylogeny of the genus *Solanum* (Solanaceae). Systematic Botany 32: 445–463. doi: 10.1600/036364407781179671

[B42] Yanosky A (2013) Paraguay'S, challenge of conserving natural habitats and biodiversity with global markets demanding for products. In: Sodhi NS Gibson L Raven PH (Eds) Conservation biology: voices from the tropics. Wiley and Sons, London, 113–119. doi: 10.1002/9781118679838.ch14

